# Adenovirus type 5 *E1A*-induced apoptosis in COX-2-overexpressing breast cancer cells

**DOI:** 10.1186/bcr1739

**Published:** 2007-07-05

**Authors:** Takeshi Sugimoto, Chandra Bartholomeusz, Ana M Tari, Naoto T Ueno

**Affiliations:** 1 Breast Cancer Translational Research Laboratory, The University of Texas M. D. Anderson Cancer Center, Houston, TX, USA; 2 Department of Stem Cell Transplantation and Cellular Therapy, The University of Texas M. D. Anderson Cancer Center, Houston, TX, USA; 3 Department of Experimental Therapeutics, The University of Texas M. D. Anderson Cancer Center, Houston, TX, USA; 4 Department of Breast Medical Oncology, The University of Texas M. D. Anderson Cancer Center, Houston, TX, USA

## Abstract

**Introduction:**

Suppression of Bcl-2 expression can overcome cellular resistance to apoptosis induced by the adenovirus type 5 gene *E1A *in models of ovarian and breast cancer. Celecoxib, a cyclooxygenase-2 (COX-2) inhibitor, is known to downregulate Bcl-2 expression. We hypothesized that celecoxib would enhance *E1A*-induced apoptosis by suppressing Bcl-2 through suppressing COX-2 expression. If successful, this strategy could represent a means of overcoming resistance to *E1A *gene therapy.

**Methods:**

We first established the cytotoxicity of celecoxib in two COX-2-overexpressing *E1A*-transfected breast cancer cell lines (MDA-MB-231 and MDA-MB-435) and in two low-COX-2-expressing *E1A*-transfected cell lines (MCF-7 (breast cancer) and SKOV3.ip1 (ovarian cancer)). We next tested whether higher sensitivity to celecoxib among these cell lines resulted from increased apoptosis by flow cytometry and western blotting. We further investigated whether suppression of Bcl-2 by celecoxib was involved in the apoptosis resulting from celecoxib treatment, and we explored whether the celecoxib-induced apoptosis in these cells depends on a COX-2 downstream pathway.

**Results:**

The two COX-2-overexpressing cell lines MDA-MB-231-*E1A *and MDA-MB-435-*E1A *were more sensitive to celecoxib than the corresponding control cells, but the two low-COX-2-expressing cell lines MCF-7-*E1A *and SKOV3.ip1-*E1A *were no more sensitive than control cells to celecoxib. Therefore, we used the MDA-MB-231-*E1A *and MDA-MB-435-*E1A *cells for all further experiments. In both cell lines, sub-G_1 _fraction was increased, or cleavage of PARP and caspase-9 were increased after 5 days of exposure to 40 μM celecoxib. However, Bcl-2 was suppressed only in the MDA-MB-435-*E1A *cells and not in the MDA-MB-231-*E1A *cells. Restoring Bcl-2 expression in the MDA-MB-435-*E1A *stable transfectants did not affect their sensitivity to celecoxib. However, adding prostaglandin E_2 _(PGE_2_) or PGF_2α _blunted the sensitivity to celecoxib of both *E1A *stable transfectants.

**Conclusion:**

We speculate that one mechanism by which celecoxib enhances *E1A*-induced apoptosis in cells that express high levels of COX-2 is through blocking PGE_2 _or PGF_2α_.

## Introduction

The adenovirus type 5 gene *E1A *is being developed as a therapeutic agent for breast, head and neck, and ovarian cancer [1-3]. The tumor-suppressive effect of *E1A *results from its induction of apoptosis, its inhibition of invasion and metastasis, and its suppression of proliferation [4]. Although the mechanism by which *E1A *induces apoptosis is not completely understood, accumulating evidence suggests that *E1A *exerts its apoptotic effect through several pathways. One such pathway involves binding of E1A to the retinoblastoma protein, which results in the release and activation of transcription factor E2F [4-6]. Activated E2F can induce apoptosis through p53-dependent and p53-independent pathways [7-9]. *E1A *is also known to induce apoptosis through p53-dependent and p53-independent pathways [10]. Several factors that modulate *E1A*-induced apoptosis have been reported, including tumor necrosis factor α (TNF-α) [11,12], TNF-related apoptosis-inducing ligand (TRAIL) [13] or TRAIL accompanied by caspase activation [14], activation of the pro-apoptotic factor p38 or inactivation of Akt/PKB [15], inhibition of nuclear factor-kappa B [16], and suppression of the Axl-Gas6 interaction [17].

However, E1A does not induce apoptosis efficiently in all types of cancer cells. The reason for this is unclear but could reflect the existence of a resistance mechanism or the oncogenic effects of the *E1A *gene. We recently showed that resistance to *E1A *gene therapy in an ovarian cancer xenograft model could be overcome by downregulating Bcl-2 with a Bcl-2 antisense oligonucleotide [18]. Bcl-2 inhibits apoptosis by inhibiting the release of cytochrome c and the activation of caspase-9 in *E1A*-transfected cells. Bcl-2 was the only major mechanism blocking E1A-induced apoptosis in our previous model. Theoretically, a means of downregulating Bcl-2 would overcome resistance to the apoptosis induced by *E1A *gene therapy. Because the US Food and Drug Administration has yet to approve Bcl-2 antisense oligonucleotide for clinical use, we are exploring other drugs that can also downregulate Bcl-2. Recent reports that cyclooxygenase-2 (COX-2) inhibitors could induce apoptosis through Bcl-2 downregulation led us to consider the role of COX-2 inhibitors in the *E1A*-induced apoptosis of cancer cells. In those reports, the selective COX-2 inhibitor NS-398 was found to downregulate Bcl-2 in LNCaP prostate cancer cells [19]; another COX-2 inhibitor, celecoxib, downregulated Bcl-2 in K562 chronic myeloid leukemia cells [20] and in MPP89 malignant mesothelioma cells [21]. *In vivo *investigations have shown that Bcl-2 downregulation by COX-2 inhibitors is accompanied by downregulation of the COX-2 protein. In one study of mice implanted with the hepatoma cell line H22, treatment with the COX-2 inhibitor nimesulide led to reductions in both COX-2 and Bcl-2 expression [22]. Similarly, celecoxib also reduced both COX-2 and Bcl-2 expression in an MTag mouse model of breast cancer [23]. Conversely, another group has proposed that COX-2 overexpression increases resistance to apoptosis through the upregulation of Bcl-2 [24]. Still others have shown that forced COX-2 overexpression or treatment with prostaglandins induces Bcl-2 expression [25,26]. Collectively, these reports suggest that COX-2 is upstream of Bcl-2 and led us to propose that suppressing COX-2 expression with the COX-2 inhibitor celecoxib will suppress Bcl-2 expression, thereby enhancing *E1A*-induced apoptosis. If successful, this strategy could represent a means of overcoming resistance to *E1A *gene therapy.

## Materials and methods

### Cell lines and reagents

Three human breast cancer cell lines (MDA-MB-231, MDA-MB-435, and MCF-7) and one ovarian cancer cell line (SKOV3.ip1, a subline of SKOV3 cells) were maintained in Dulbecco's modified Eagle's medium/Ham's F-12 medium (DMEM/F12; Gibco-BRL, Grand Island, NY, USA) supplemented with 10% fetal bovine serum and penicillin/streptomycin, and maintained in a humidified atmosphere of 5% CO_2 _at 37°C. The parental cell lines, the vector only transfection control cells, and the *E1A*-transfected cells were all kindly provided by Dr Mien-Chie Hung (The University of Texas M D Anderson Cancer Center, Houston, TX, USA) [15,27,28]. The *E1A *stable transfectants were selected by growing them in DMEM/F12 medium containing 500 μg/ml G418. All experiments were conducted under the guidelines of the M D Anderson Cancer Center.

Celecoxib (LKT Laboratories, St Paul, MN, USA) was dissolved in dimethylsulfoxide to 100 mM and stored at -20°C. For the cell culture experiments, celecoxib was diluted with DMEM/F12 in various concentrations. The final concentration of dimethylsulfoxide in the DMEM/F12 medium was kept at less than 0.1%. PGE_2 _and PGF_2α _(Cayman Chemical, Ann Arbor, MI, USA) were dissolved in dimethlysulfoxide to 10 mM and stored at -20°C.

### Cell viability assays

Cell viability was assessed with 3-(4,5-dimethylthiazol-2-yl)-2,5-diphenyltetrazolium bromide (MTT) [29] and trypan blue exclusion assays. For the MTT assay, cells were plated in 96-well plates (1.2 × 10^4 ^cells/well for MDA-MB-231 cells or 8.0 × 10^3 ^cells/well for MDA-MB-435, MCF-7, and SKOV3.ip1 cells) in 80 μl of medium and incubated for 24 h. Then, 20 μl of fresh medium containing celecoxib at 0–300 μM were added to each well, resulting in final celecoxib concentrations of 0–60 μM in five sequential dilutions. After cells had been cultured with celecoxib for 3 or 5 days, MTT (Sigma Chemical Co., St Louis, MO, USA) was added to a final concentration of 1 mg/ml. Reaction mixtures were incubated for 3 h. The resulting crystals were dissolved in dimethlysulfoxide (200 μl), and optical density was measured at 570 nm with a microplate reader (Bio-Rad Laboratories, Hercules, CA, USA).

We also used a trypan blue exclusion assay to confirm cell viability during treatment with 40 μM celecoxib, because the viability varied greatly among cell types. For that assay, MDA-MB-231 cells (4 × 10^5^) or MDA-MB-435 cells (2 × 10^5^) were plated in six-well plates in 2 ml of DMEM/F12 and incubated for 24 h. Then, 500 μl of fresh medium containing 200 μM celecoxib was added to each well, resulting in a final concentration of 40 μM, and the cells were incubated for 5 days. To determine the effect of PGE_2 _or PGF_2α _on celecoxib sensitivity, exogenous PGE_2 _or PGF_2α _(10 μM) was added to the celecoxib solution. After being cultured with celecoxib in the presence or absence of PGE_2 _or PGF_2α _for 5 days, cells were harvested by trypsinization and incubated with 0.4% trypan blue (Sigma). Cell viability was calculated as the percentage of viable (nonstaining) cells.

### Flow cytometry

For flow-cytometric analysis of apoptosis, cells were harvested by trypsinization, washed twice with ice-cold PBS, and fixed with cold 70% ethanol at -20°C overnight. The fixed cells were washed twice with PBS and suspended in 1 ml of PBS containing Tween-20 (0.5%), RNase (10 μg/ml), and propidium iodide (10 μg/ml). The sub-G_1 _(apoptotic) cell population was measured with a FACScan cytofluorometer (Becton Dickinson, San Jose, CA, USA).

### Western blotting

For western blot analyses, cells were washed with PBS and lysed in lysis buffer (20 mM Na_2_PO_4_, 150 mM NaCl, 1% Triton X-100, 1% aprotinin, 1 mM phenylmethylsulfonyl fluoride, 100 mM NaF, and 2 mM Na_3_VO_4_) as described previously [18]. Proteins were separated by polyacrylamide gel electrophoresis on a sodium dodecyl sulfate-polyacrylamide gel and transferred to a polyvinylidene difluoride membrane (Bio-Rad). Membranes were incubated with primary antibodies specific for COX-2 (1:1000; Cayman Chemical); Bcl-2 (1:500) and E1A (1:500) (both from BD PharMingen); poly-ADP ribosome polymerase (PARP) (1:1000) and cleaved caspase-9 (1:200) (both from Cell Signaling Technology, Beverly, MA, USA); caspase-8 (1:200) (from Oncogene Research Products, San Diego, CA, USA); and actin (1:5000) (Sigma). Then, membranes were incubated with fluorescent-conjugated mouse (1:5000) or rabbit (1:5000) secondary antibodies (IRdye; Li-Cor Biosciences, Lincoln, NE, USA). The membranes were scanned and relative protein expression levels estimated by using an Odyssey western blotting system (Li-Cor Biosciences). Downregulation was defined as a protein expression level at least 20% less than that of the control (untreated) cells.

### Bcl-2 transfections

The Bcl-2 expression vector was constructed by inserting Bcl-2 cDNA at the *Eco*RI site of the pCl-neo mammalian expression vector (Promega, Madison, WI, USA). Briefly, MDA-MB-435 cells (4 × 10^5^) were cultured for 24 h in six-well plates in 1 ml/well of DMEM/F12 with 10% fetal bovine serum until 60–70% confluence. The next day, the liposomal vector of the SN gene delivery system [30] was incubated with Bcl-2 DNA at a ratio of 4 μg DNA: 5 μl SN in 100 μl Opti-MEM in each well and added to the cultures. Bcl-2-overexpressing cells and control cells were plated 24 h later and tested for viability with a trypan blue exclusion assay as described above.

### Prostaglandin analyses

MDA-MB-231-*E1A *cells (4 × 10^5^) or MDA-MB-435-*E1A *cells (2 × 10^5^) were plated in six-well plates in 2 ml of DMEM/F12, incubated for 24 h, and treated with celecoxib (0–40 μM) for 120 h. At that time, cellular supernatants were collected and analyzed for PGE_2 _and PGF_2α _by enzyme-linked immunosorbent assay (Cayman Chemical) according to the manufacturer's instructions.

### Statistical analyses

Two-tailed paired *t*-tests were used to compare data between groups. *P *< 0.05 was considered to be statistically significant. Data were expressed as means ± SD of at least three independent experiments, each of which was run in quadruplicate.

## Results

### COX-2 expression in E1A-transfected breast and ovarian cancer cell lines

**Figure 1:**
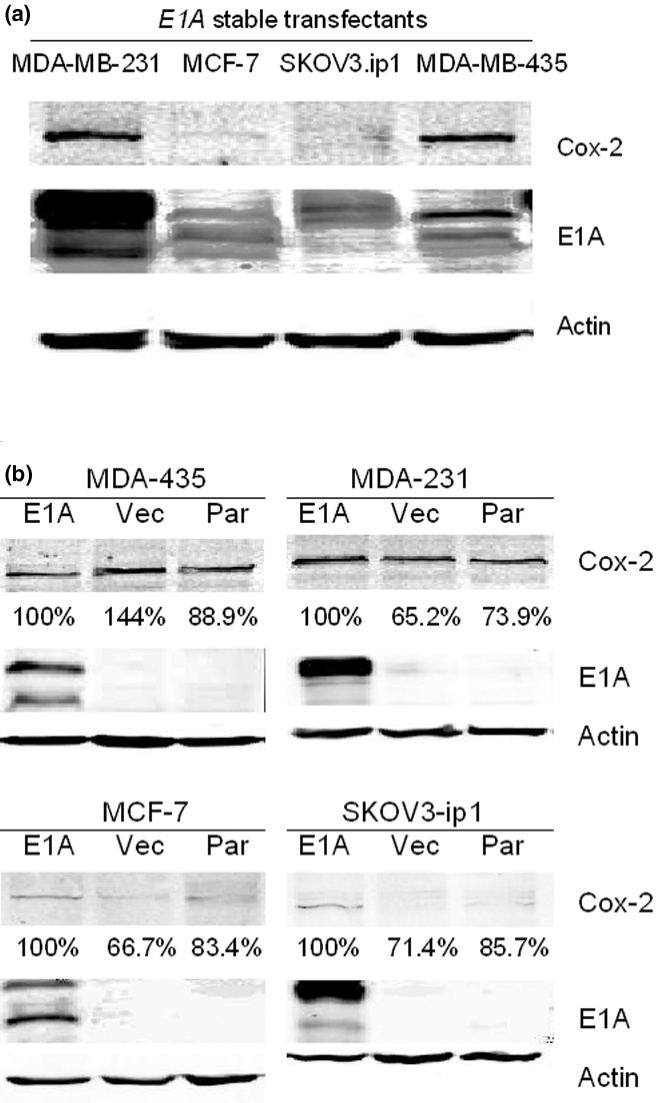
COX-2 protein expression in breast and ovarian cancer cell lines stably transfected with *E1A*. **(a) **MDA-MB-231-*E1A *stable transfectants produced the greatest amounts of COX-2; MDA-MB-435-*E1A *cells produced 78% of that amount, but the SKOV3.ip1-*E1A *and MCF-7-*E1A *cells produced only 6% of that amount. MDA-MB-231-*E1A *cells expressed slightly more E1A than the other three cell lines. **(a) **COX-2 protein expression level between the *E1A *stable transfectants and their corresponding vector control cells or parent cells. If the COX-2 expression levels of each *E1A *transfectant is defined as 100%, the corresponding COX-2 expression levels of the vector controls were as follows: 65% for MDA-MB-231, 144% for MDA-MB-435, 71% for SKOV3.ip1 and 67% for MCF-7.

We first investigated the expression of COX-2 protein in three breast cancer cell lines (MDA-MB-231, MDA-MB-435, and MCF-7) and an ovarian cancer cell line (SKOV3.ip1), all stably transfected with *E1A*, to determine the relationship between COX-2 protein expression level and sensitivity to celecoxib. Western blot analyses showed that COX-2 expression was highest in the MDA-MB-231-*E1A *transfectants, followed closely by the MDA-MB-435-*E1A *transfectants. By contrast, COX-2 expression was low in the SKOV3.ip1-*E1A *and MCF-7-*E1A *transfectants (Figure 1A). If the COX-2 expression level of the MDA-MB-231-*E1A *stable transfectants is considered to be 100%, the relative percentage COX-2 expression for each stable transfectant is as follows: MDA-MB-435-*E1A*, 78%; SKOV3.ip1-*E1A*, 6%; and MCF-7-*E1A*, 6%. Thus, we defined MDA-MB-231-*E1A *and MDA-MB-435-*E1A *stable transfectants as high-COX-2-expressing cell lines, and SKOV3.ip1-*E1A *and MCF-7-*E1A *stable transfectants as low-COX-2-expressing cell lines.

To rule out the possibility that *E1A *transfection affects COX-2 expression levels, we compared COX-2 levels in *E1A *stable transfectants with that in the corresponding vector control cells by western blotting analysis. If the COX-2 expression levels of each *E1A *transfectant is defined as 100%, the corresponding COX-2 expression levels of the vector controls were as follows: 65% for MDA-MB-231, 144% for MDA-MB-435, 71% for SKOV3.ip1 and 67% for MCF-7 (Figure 1B). Thus, we found that *E1A *transfection did not consistently affect COX-2 expression levels in these cell lines.

### Celecoxib induces apoptosis in MDA-MB-231-*E1A *and MDA-MB-435-*E1A *stable transfectants

**Figure 2:**
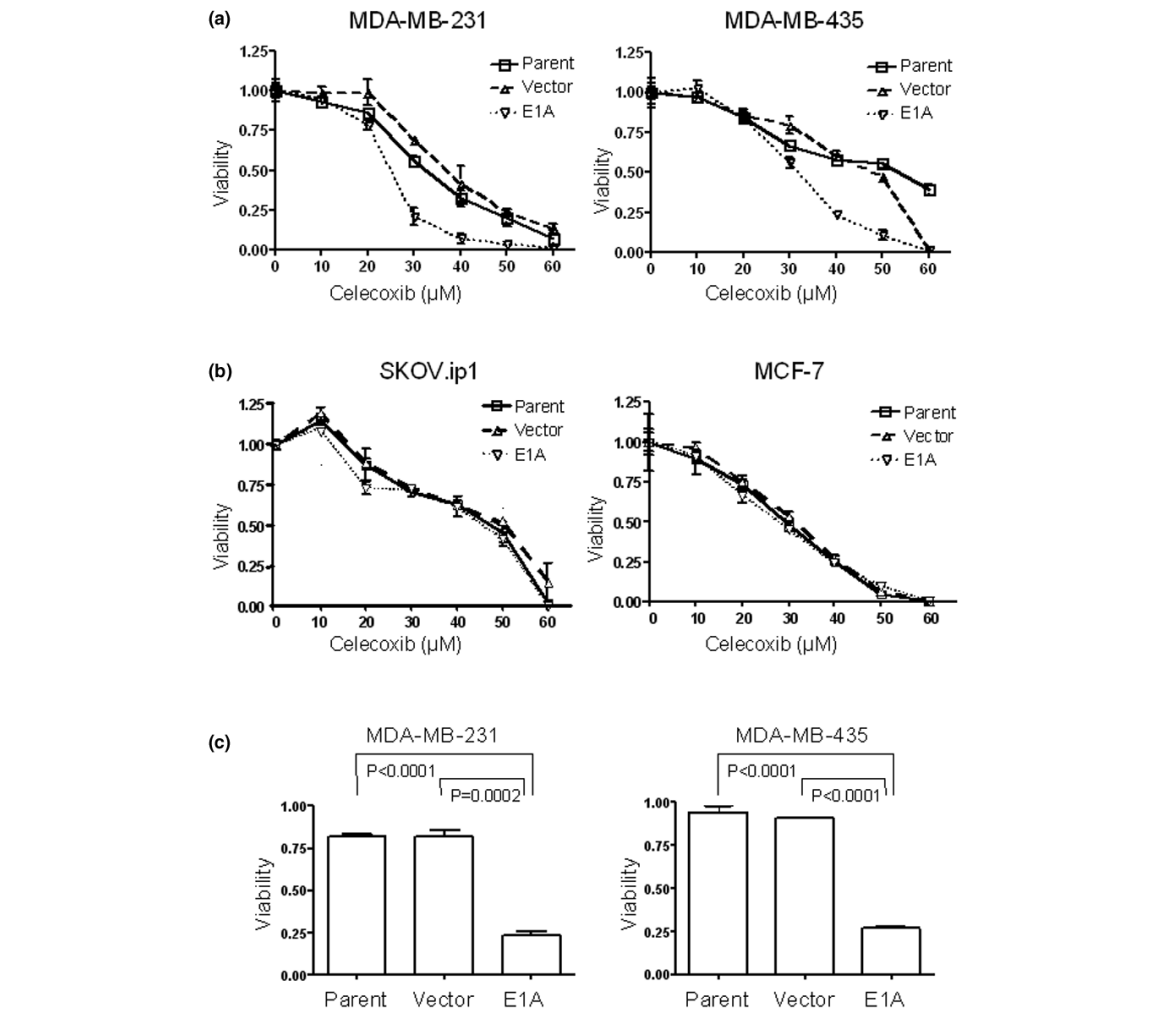
Celecoxib decreases the viability of MDA-MB-231-*E1A *and MDA-MB-435-*E1A *stable transfectants. **(b) **MTT assays after a 5-day exposure to 0–60 μM celecoxib indicate substantial reductions in cell viability in all variants of the MDA-MB-231 and MDA-MB-435 cell lines (*E1A *stable transfectants, vector control, and parental control cells). Each point represents means from tests performed in quadruplicate; the bars are standard deviations. In both cell lines, the *E1A *transfectants were more sensitive than the vector control or parental control cells. **(b) **MTT assays in all variants of the SKOV3.ip1 and MCF-7 cell lines. The *E1A *transfectants have no difference to sensitivity for celecoxib in both cell lines. **(c) **Trypan blue assays after a 5-day exposure to 40 μM celecoxib show substantial reductions in viability of MDA-MB-231 and MDA-MB-435 cells. Values shown are normalized to the viability of the control (untreated) cells. Each bar represents means from tests performed in quadruplicate; bars are standard deviations. *P *values are from two-tailed paired *t *tests.

After establishing the relative amounts of COX-2 protein expressed by the *E1A *stable transfectants, we investigated whether those transfectants were more sensitive to celecoxib than their respective controls by using an MTT assay. The high-COX-2-expressing MDA-MB-231-*E1A *and MDA-MB-435-*E1A *cells were more sensitive to celecoxib (after 5 days of exposure to 0–60 μM) than the vector control or parental control cells (Figure 2A). By contrast, the low-COX-2-expressing cells (SKOV3.ip1 and MCF-7) showed no difference in celecoxib sensitivity between the *E1A *transfectants and the controls (Figure 2B).

**Figure 3:**
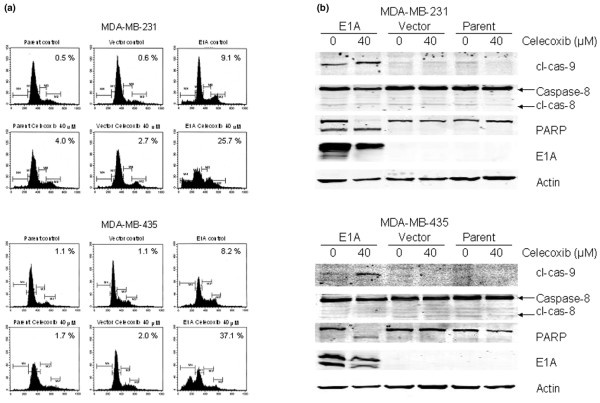
Celecoxib enhances apoptosis of MDA-MB-231-*E1A *and MDA-MB-435-*E1A *stable transfectants. **(a) **Cell cycle distribution of MDA-MB-231-*E1A *and MDA-MB-435-*E1A *cells was detected by fluorescence-activated cell sorting after a 5-day exposure to 0 or 40 μM celecoxib. The percentage of cells in sub-G_1 _(apoptosis) appears at the upper right of each graph. **(b) **Western blots of MDA-MB-231 and MDA-MB-435 cells treated with 0 or 40 μM celecoxib for 5 days and tested for cleaved caspase-9 (cl-cas-9), uncleaved and cleaved caspase-8 (cl-cas-8), PARP (uncleaved and cleaved), E1A, and actin. Cleaved PARP and cleaved caspase-9 levels were higher after celecoxib treatment in the MDA-MB-231-*E1A *and MDA-MB-435-*E1A *stable transfectants, but expression of cleaved caspase-8 (cl-cas-8) did not change.

To quantitatively compare differences in sensitivity to celecoxib among cells, we tested the MDA-MB-231-*E1A *and MDA-MB-435-*E1A *transfectants and their controls with a trypan blue assay. After a 5-day exposure to 40 μM celecoxib, the MDA-MB-231-*E1A *cells were significantly more sensitive (mean 23.8% ± SD 2.2% viable cells) than the MDA-MB-231 vector control cells (82.3% ± 3.9%) (*P *= 0.0002) or the MDA-MB-231 parental cells (82.0% ± 1.4%) (*P *< 0.0001). Similarly, the MDA-MB-435-*E1A *stable transfectants were more sensitive to celecoxib (27.2% ± 1.2% viable cells) than the MDA-MB-435 vector control cells (91.2% ± 0.5%) (*P *< 0.0001) or the MDA-MB-435 parental cells (93.9% ± 3.5%) (*P *< 0.0001) (Figure 2C). We then investigated whether these differences in viability had been caused by apoptosis by using flow cytometry. A 5-day exposure to 40 μM celecoxib increased the sub-G_1 _fraction from 9.1% to 25.7% in MDA-MB-231-*E1A *cells and from 8.2% to 37.1% in MDA-MB-435-*E1A *cells (Figure 3A), indicating increases in apoptosis. We also tested cleavage of PARP, caspase-8, and caspase-9 as other indicators of apoptosis. Celecoxib treatment led to the disappearance of uncleaved PARP (116 kDa) in both cell lines; it also led to the appearance of cleaved PARP (89 kDa) in MDA-MB-435-*E1A *cells and the appearance of cleaved PARP expression in MDA-MB-231-*E1A *cells (Figure 3B). Celecoxib treatment also led to increases in cleaved caspase-9 (37 kDa) levels in both MDA-MB-231-*E1A *and MDA-MB-435-*E1A *transfectants but did not affect levels of cleaved caspase-8 (28 kDa). These results suggest that celecoxib treatment (5 days at 40 μM) induced apoptosis in MDA-MB-231 and MDA-MB-435 cells stably transfected with *E1A*.

### Celecoxib downregulates COX-2 protein expression in MDA-MB-231 and MDA-MB-435 cells

**Figure 4:**
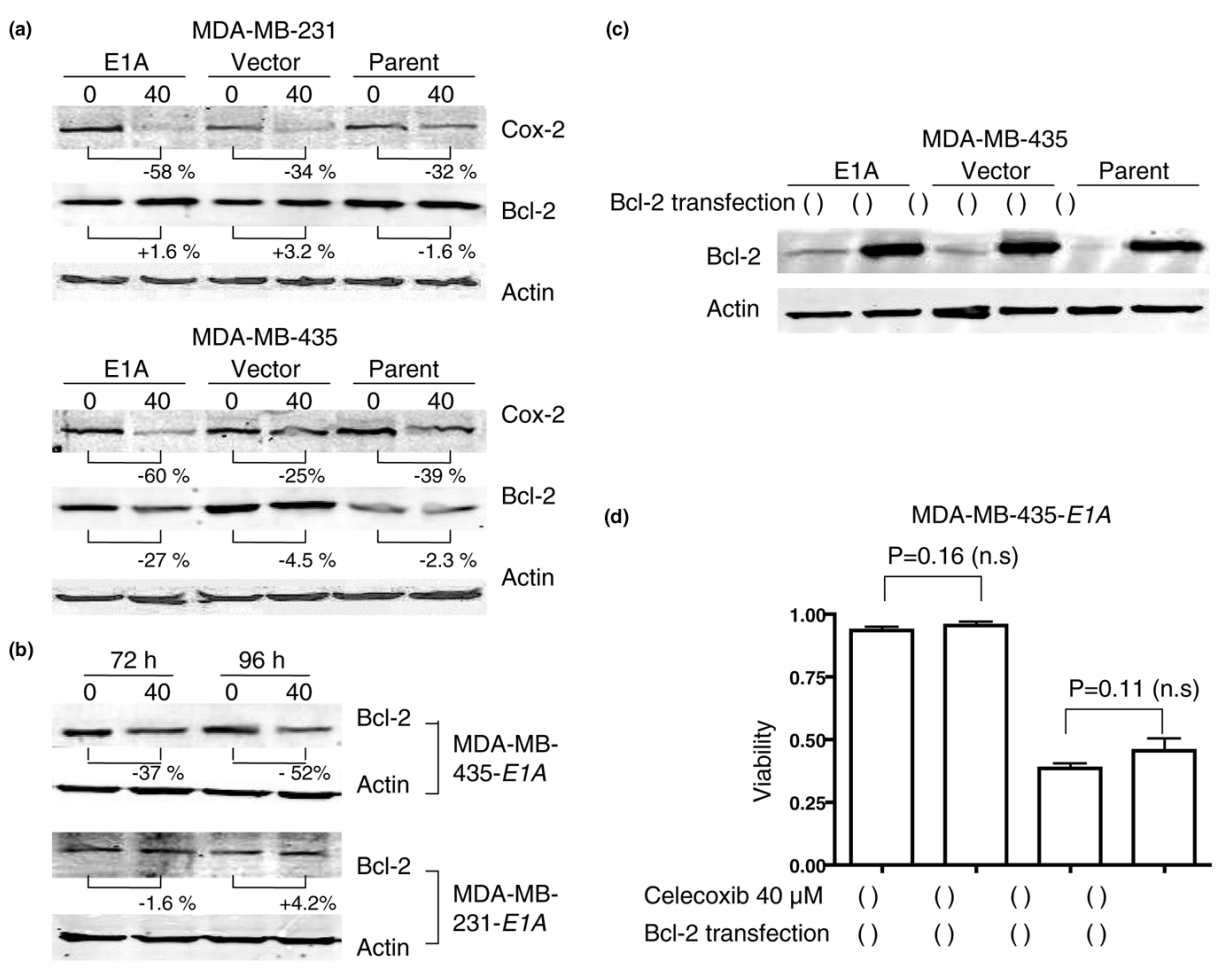
Celecoxib downregulated COX-2 protein expression in all MDA-MB-231 and MDA-MB-435 variants, but celecoxib downregulated Bcl-2 expression in only the MDA-MB-435-*E1A *stable transfectants. (**a**) Western blots of MDA-MB-231 and MDA-MB-435 cells treated with 0 or 40 μM celecoxib for 5 days and tested for COX-2 and Bcl-2. Percentages indicate differences relative to the 0 μM control samples. Protein expression was considered to be downregulated if the treated condition was at least 20% less than the control (untreated) condition. (**b**) Time course of Bcl-2 expression after treatment with 0 or 40 μM celecoxib in MDA-MB-435-*E1A *and MDA-MB-231-*E1A *stable transfectants. Bcl-2 was suppressed at both 72 and 96 h in the MDA-MB-435-*E1A *stable transfectants but was not suppressed in the MDA-MB-231-*E1A *stable transfectants. (**c**) Transfection of MDA-MB-435 cells with Bcl-2 DNA (+) or a control DNA (-) led to overexpression of Bcl-2 in all variants. (**d**) MDA-MB-435-*E1A *cells made to overexpress Bcl-2 and non-Bcl-2-overexpressing cells were treated with 0 or 40 μM celecoxib for 5 days, and cell viability was determined with a trypan-blue assay. Bcl-2 overexpression did not restore sensitivity to celecoxib (*P *= 0.11).

Evidence that nonsteroidal anti-inflammatory drugs can suppress COX-2 by transcriptional regulation [31] as well as suppressing both COX-2 and Bcl-2 protein expression [22,23] led us to investigate whether celecoxib would change the amount of COX-2 protein expressed by MDA-MB-231 and MDA-MB-435 cells. Indeed, COX-2 protein expression was downregulated in all MDA-MB-231 cell variants; the percentage decreases were 32% for the MDA-MB-231 parental cells, 34% for the vector control cells, and 58% for the *E1A *stable transfectants (Figure 4A). COX-2 protein expression was also decreased in all MDA-MB-435 variants, with the percentages being 39% for MDA-MB-435 parental cells, 25% for the vector control cells, and 60% for the *E1A *stable transfectants (Figure 4A). In both MDA-MB-231 and MDA-MB-435 cells, COX-2 was suppressed to a greater extent in the *E1A *transfectants than in the corresponding parental or vector control cells. These results suggest that celecoxib-induced apoptosis in cells expressing E1A involves the suppression of COX-2.

### Bcl-2 suppression does not contribute to celecoxib-induced apoptosis in *E1A *stable transfectants

Next, we investigated whether suppression of Bcl-2 by celecoxib is involved in the apoptosis resulting from celecoxib treatment. After a 5-day treatment with 40 μM celecoxib, Bcl-2 levels in the MDA-MB-435 cells were decreased (by 27%) only in the *E1A *transfectant; Bcl-2 level did not change in any of the MDA-MB-231 variants (Figure 4A). In timecourse experiments with the same celecoxib concentration (40 μM), Bcl-2 was suppressed by 37% at 72 h and by 52% at 96 h in MDA-MB-435-*E1A *cells. However, Bcl-2 was not suppressed at either measurement time in the MDA-MB-231-*E1A *cells (Figure 4B).

As a further step in determining the contribution of Bcl-2 suppression to celecoxib-induced apoptosis, we transfected Bcl-2 DNA into the MDA-MB-435 variants (Figure 4C) to see if restoring Bcl-2 expression would affect sensitivity to celecoxib. Bcl-2 restoration did not affect the viability of MDA-MB-435-*E1A *stable transfectants after a 5-day treatment with 40 μM celecoxib (Figure 4D). These results suggest that celecoxib induces apoptosis in MDA-MB-231-*E1A *and MDA-MB-435-*E1A *stable transfectants regardless of Bcl-2 expression.

### Celecoxib enhances apoptosis of MDA-MB-231-*E1A *and MDA-MB-435-*E1A *cells via prostaglandins E_2 _or F_2α_

**Figure 5:**
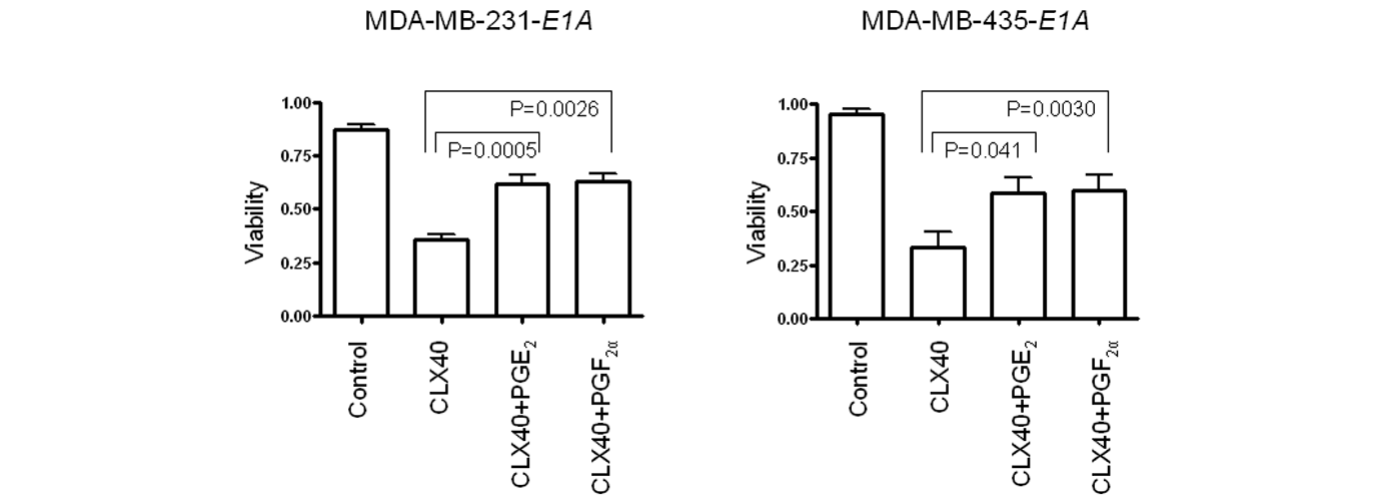
Celecoxib-induced apoptosis of MDA-MB-231-*E1A *and MDA-MB-435-*E1A *cells depends on PGE_2 _or PGF_2α_. Treatment of MDA-MB-231-*E1A *and MDA-MB-435-*E1A *stable transfectants with 0 or 40 μM celecoxib (CLX) plus 10 μM of either PGE_2_or PGF_2α _for 5 days blunted sensitivity to celecoxib in both cell lines.

Given our findings that celecoxib induced apoptosis in the *E1A *stable transfectants and that COX-2 downregulation is involved in this apoptosis but Bcl-2 suppression is not, we next explored whether the celecoxib-induced apoptosis in these cells depends on a pathway downstream of COX-2. For these experiments, we tested the effects of a 5-day treatment with 40 μM celecoxib on cell viability with or without the addition of 10 μM prostaglandin (PG) E_2 _or PGF_2α_, two molecules located downstream of COX-2. In the MDA-MB-231-*E1A *cells, treatment with celecoxib alone produced a mean of 35.9% viable cells (± 2.7% SD); the addition of either prostaglandin substantially improved cell viability (62.1% ± 3.9% viable cells for PGE_2 _(*P *= 0.0005), 63.0% ± 3.8% for PGF_2α _(*P *= 0.0026). Results were similar for the MDA-MB-435-*E1A *cells (33.3% ± 7.5% for celecoxib only, 58.6% ± 7.7% for PGE_2 _(*P *= 0.041), and 60.2% ± 6.7% for PGF_2α _(*P *= 0.0030)) (Figure 5). These results suggest that celecoxib enhances apoptosis of cells that stably express *E1A *in part by blocking PGE_2 _or PGF_2α_.

To test the effect of celecoxib on prostaglandin synthesis, we assessed PGE_2 _and PGF_2α _levels in MDA-MB-231-*E1A *and MDA-MB-435-*E1A *cells treated for 5 days with 40 μM celecoxib (data not shown). Enzyme-linked immunosorbent assay showed that in the MDA-MB-231-*E1A *cells, celecoxib treatment significantly inhibited PGE_2 _(mean ± SD 0.20 pg/ml ± 0.08 pg/ml vs 2.16 pg/ml ± 1.25 pg/ml for dimethylsulfoxide control; *P *= 0.002), but celecoxib had no effect on PGE_2 _synthesis in the MDA-MB-435-*E1A *cells. Conversely, celecoxib inhibited PGF_2α _in MDA-MB-435-*E1A *cells (121.7 pg/ml ± 12.3 pg/ml vs 173.6 pg/ml ± 7.4 pg/ml for control, *P *= 0.003) but did not affect PGF_2α _synthesis in MDA-MB-231-*E1A *cells.

## Discussion

Our hypothesis was that celecoxib would enhance *E1A*-induced apoptosis by suppressing COX-2 expression and thereby suppressing Bcl-2 expression. In exploring the possibility that the COX-2 inhibitor celecoxib would downregulate Bcl-2, we found that celecoxib did enhance *E1A*-induced apoptosis in cells that express high levels of COX-2 protein. We also found that PGE_2 _or PGF_2α _are involved in this apoptotic pathway. However, celecoxib-induced apoptosis did not depend on suppression of Bcl-2.

With regard to suppression of Bcl-2 by COX-2 inhibitors, some previous studies have shown that the ability of COX-2 inhibitors to induce apoptosis in cancer cells depends on the downregulation of Bcl-2 [19-23]. However, others have reported that COX-2 inhibitors can induce apoptosis in cancer cells independently of Bcl-2 [29,32]. Cao and Prescott [33] proposed that Bcl-2 overexpression is probably caused by reductions in arachidonic acid and increases in PGE_2 _levels. Nevertheless, our finding that COX-2 suppression did not suppress Bcl-2 leads us to propose that other mechanisms exist by which celecoxib induces apoptosis, at least in the breast cancer cell lines we tested. In other words, it is unclear how much suppression of Bcl-2 is enough to induce significant apoptosis, or, indeed, if celecoxib has the capacity to downregulate Bcl-2. It is possible that only the reduction in Bcl-2 led to apoptosis in the MDA-MB-435-*E1A *stable transfectants; however, that reduction would have to have been substantial. We showed that 40 μM of celecoxib was enough to enhance *E1A*-induced apoptosis in MDA-MB-231-*E1A *and MDA-MB-435-*E1A *stable transfectants regardless of Bcl-2 suppression. This result suggests that other molecules than Bcl-2 could be critical for celecoxib to enhance *E1A*-induced apoptosis.

We then investigated the involvement of PGE_2 _or PGF_2α_, two molecules located downstream of COX-2. The synthesis of prostaglandins is known to depend on COX-2 activity. In one study, the synthesis of PGE_2 _or PGF_2α _was enhanced by the overexpression of COX-2 in the mammary glands of COX-2 transgenic mice [34]. In addition, PGF_2α _has been shown to promote tumorigenesis in endometrial cancer cells [35]. Several reports indicate that the reduction of COX-2 inhibits the release of prostaglandins. For example, treatment of the COX-2-overexpressing myeloma cell line ARH-77 with indomethacin led to the reduction of PGD_2_, PGE_2_, and PGF_2α _[36]. Celecoxib has been shown to inhibit the release of PGE_2 _or PGF_2α _from ureteral segments in swine [37]. NS-398 inhibited the production of prostaglandins, including PGD_2_, PGE_2 _and PGF_2α_, in the prostate cancer cell line PC-3 [38]. We speculate that both PGE_2 _and PGF_2α _derived from COX-2 could be key factors in modulating the apoptotic effect in *E1A*-transfected cell lines. Indeed, we found that blocking PGE_2 _and PGF_2α _production was crucial for celecoxib-induced apoptosis in the *E1A *stable transfectants. We confirmed here that both PGE_2 _and PGF_2α _influenced apoptosis, but celecoxib suppressed different prostaglandins in the two different cell lines. This difference could reflect the involvement of other pathways that modulate apoptosis in E1A-transfected cell lines.

Davis *et al*. [39] reported that the COX-2 inhibitor NS-398 was more cytotoxic in a prostate epithelial cell line in which E2F1 had been activated than in the original prostate epithelial cell lines, speculating that the reason for the greater cytotoxicity was a disruption in the retinoblastoma/E2F complexes. We showed that caspase-9 and PARP were activated, but caspase-8 was not activated, in celecoxib-treated MDA-MB-231-*E1A *and MDA-MB-435-*E1A *stable transfectants (Figure 3B). These findings are consistent with others showing that celecoxib enhances caspase-3 or caspase-9 activation through PGE_2 _inhibition [42,43]. We speculate that PGE_2 _or PGF_2α _(or both) inhibit intrinsic apoptotic pathway induced by *E1A*. This speculation is, to the best of our knowledge, the first to link PGE_2 _or PGF_2α _in the apoptosis associated with *E1A*.

Generally speaking, COX-2 inhibitors, including celecoxib, are thought to block prostaglandin synthesis by inhibiting the enzymatic activity of COX-2. However, COX-2 inhibitors (including celecoxib) might also act by suppressing production of the COX-2 protein [31,44,45]. We showed that the celecoxib-sensitive MDA-MB-231-*E1A *and MDA-MB-435-*E1A *stable transfectants originally overexpressed COX-2, and they produced less COX-2 protein in the presence of celecoxib (Figures 1 and 4A). Our finding that COX-2 levels were highest in the MDA-MB-231-E1A cells was confirmed by others' reports that the MDA-MB-231 cell line expresses high levels of COX-2 [46-48]. Moreover, our findings regarding sensitivity to celecoxib imply that celecoxib inhibited the production of prostaglandins not only by inhibiting COX-2 enzymatic activity but also by modulating COX-2 protein expression. A previous report indicated that modulating COX-2 protein expression with a structural analog of celecoxib (sc-236) or with the pharmacologic COX-2 protein-suppressing agent curcumin affected apoptosis in the COX-2-positive colon cancer cell line HT-29 [49]; another report showed that modulating COX-2 protein expression with the retinoid X receptor-selective retinoid LGD1069 decreased PGE_2 _production in normal human mammary epithelial cells [50]. We speculate that the modulation of COX-2 protein expression by celecoxib is a significant part of its effect in reducing prostaglandin levels.

Our study had some limitations. For one thing, although we did confirm that all four *E1A *stable transfectants expressed E1A (Figure 1), the level of expression was not uniform among the cell lines. Specifically, MDA-MB-231-*E1A *cells expressed slightly more E1A than the other three cell lines, a result that has also been shown by others [15,28]. Thus, we were not able to exclude the possibility that E1A expression level could affect sensitivity to celecoxib. In addition, we examined only one COX-2 inhibitor, celecoxib. COX-2 inhibitors other than celecoxib can downregulate Bcl-2 [19,22]; whether other COX-2 inhibitors would downregulate Bcl-2 expression to a greater extent than celecoxib remains to be seen. Elucidation of the precise mechanism by which COX-2 inhibitors downregulate Bcl-2 will help to clarify the appropriate uses of COX-2 inhibitors in preclinical settings for treating cancer.

## Conclusion

We found that celecoxib enhanced *E1A*-induced apoptosis in breast cancer cells that express high levels of COX-2 protein and that this effect depended, at least in part, on blocking the production of PGE_2 _or PGF_2α_. Further studies exploring the precise mechanism by which prostaglandins influence *E1A*-induced apoptosis, and confirmation of synergistic effects between *E1A *gene therapy and COX-2 inhibitor treatment in cancer xenograft models, are needed to establish conclusively that COX-2 inhibitors can overcome resistance to *E1A*-induced apoptosis.

## Abbreviations

COX-2 = cyclooxygenase-2; DMEM/F12 = Dulbecco's modified Eagle's medium/Ham's F-12 medium; *E1A *= adenovirus type 5 gene *E1A*; MTT = 3-(4,5-dimethylthiazol-2-yl)-2,5-diphenyltetrazolium bromide; PARP = poly ADP-ribosome polymerase; PBS = phosphate-buffered saline; PGE_2 _= prostaglandin E_2_; PGF_2α _= prostaglandin F2α; TNF-α = tumor necrosis factor-α; TRAIL = TNF-related apoptosis-inducing ligand.

## Competing interests

The authors declare that they have no competing interests.

## Authors' contributions

TS participated in conceiving and designing the study, carried out many of the experiments, and drafted the manuscript. CB participated in the collection, analysis, and interpretation of findings, provided procedural guidance and expertise, and participated in revising the manuscript. AMT, an expert in COX and COX inhibition in cancer, conceived the prostaglandin experiments. NTU led the conception and design of the study, the analysis and interpretation of the findings, and the revisions to the manuscript. All authors read and approved the final manuscript.
